# Genome-wide identification and expression pattern analysis of *MIKC-Type MADS-box* genes in *Chionanthus retusus*, an androdioecy plant

**DOI:** 10.1186/s12864-024-10569-8

**Published:** 2024-07-02

**Authors:** Maotong Sun, Dongyue Wang, Ying Li, Muge Niu, Cuishuang Liu, Laishuo Liu, Jinnan Wang, Jihong Li

**Affiliations:** 1 College of Forestry, Shandong Agricultural University, Tai’an, Shandong Province 271018 China; 2 Mountain Tai Forest Ecosystem Research Station of State Forestry and Grassland Administration, Shandong Agricultural University, Tai’an, Shandong Province 271018 China; 3 State Forestry and Grassland Administration Key Laboratory of Silviculture in Downstream Areas of the Yellow River, Tai’an, Shandong Province 271018 China

**Keywords:** Androdioecy, *Chionanthus retusus*, Floral organ, *MIKC-type MADS-box* gene

## Abstract

**Background:**

The *MADS-box* gene family is widely distributed in the plant kingdom, and its members typically encoding transcription factors to regulate various aspects of plant growth and development. In particular, the *MIKC-type MADS-box* genes play a crucial role in the determination of floral organ development and identity recognition. As a type of androdioecy plant, *Chionanthus retusus* have unique gender differentiation. Manifested as male individuals with only male flowers and female individuals with only bisexual flowers. However, due to the lack of reference genome information, the characteristics of *MIKC-type MADS-box* genes in *C. retusus* and its role in gender differentiation of *C. retusus* remain largely unknown. Therefore, it is necessary to identify and characterize the *MADS-box* gene family within the genome of the *C. retusus*.

**Results:**

In this study, we performed a genome-wide identification and analysis of *MIKC-type MADS-box* genes in *C. retusus* (2n = 2x = 46), utilizing the latest reference genome, and studied its expression pattern in individuals of different genders. As a result, we identified a total of 61 *MIKC-type MADS-box* genes in *C. retusus*. 61 *MIKC-type MADS-box* genes can be divided into 12 subfamilies and distributed on 18 chromosomes. Genome collinearity analysis revealed their conservation in evolution, while gene structure, domains and motif analysis indicated their conservation in structure. Finally, based on their expression patterns in floral organs of different sexes, we have identified that *CrMADS45* and *CrMADS60* may potentially be involved in the gender differentiation of *C. retusus*.

**Conclusions:**

Our studies have provided a general understanding of the conservation and characteristics of the *MIKC-type MADS-box* genes family in *C. retusus*. And it has been demonstrated that members of the AG subfamily, *CrMADS45* and *CrMADS60*, may play important roles in the gender differentiation of *C. retusus*. This provides a reference for future breeding efforts to improve flower types in *C. retusus* and further investigate the role of *MIKC-type MADS-box* genes in gender differentiation.

**Supplementary Information:**

The online version contains supplementary material available at 10.1186/s12864-024-10569-8.

## Background

The ornamental value and biodiversity of plants are increasingly appreciated in landscaping worldwide [1, 2]. Among the many plants used in urban landscapes, those that are cold-resistant and adaptable are particularly valuable [3]. This study aims to explore a plant with unique aesthetic and ecological value, namely the *Chionanthus retusus*. Our research not only enriches our understanding of ornamental tree species but also promotes the protection and utilization of their genetic diversity.

**Fig. 1:**
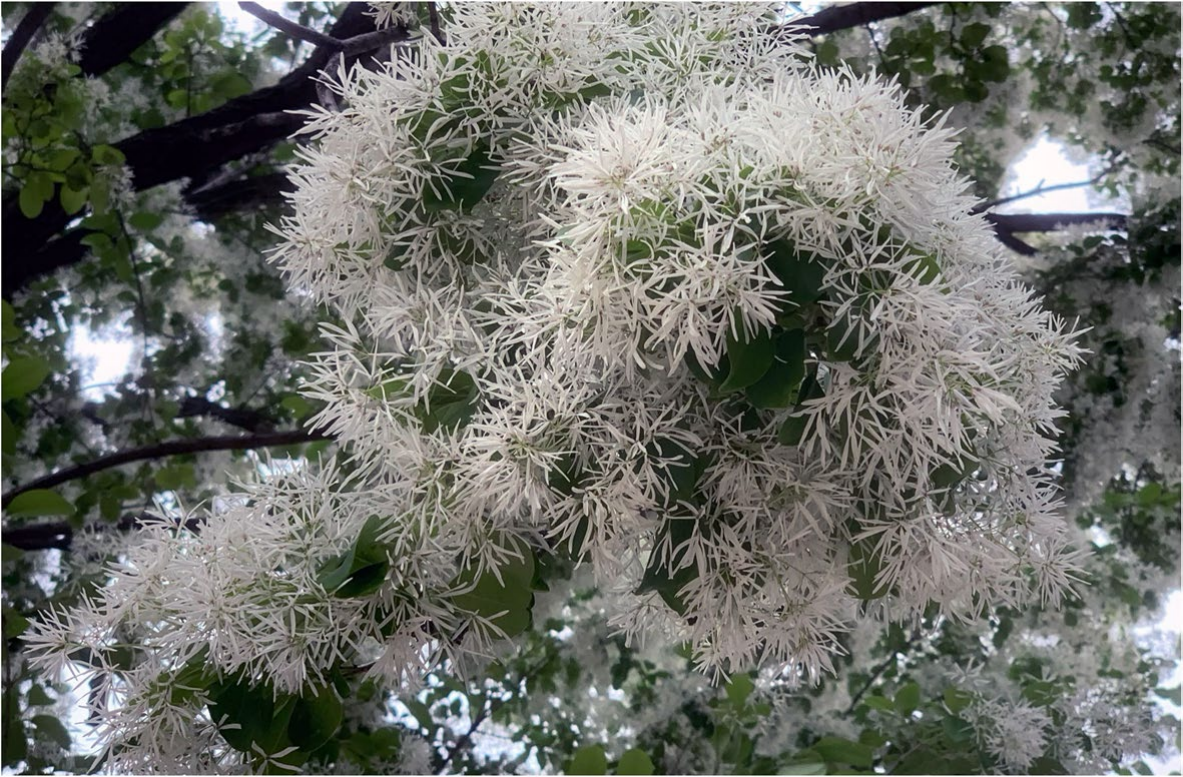
The *C. retusus* blooms in April

*C. retusus* (also known as Chinese Fringe tree), belonging to the Oleaceae family within the order Lamiales, is a true dicotyledonous plant [4]. It is valued for its unique landscape value and adaptive characteristics [5]. The Oleaceae family is a large family that includes multiple plant species, among which the *Chionanthus* genus is composed of multiple species, *C. retusus*, as one of its members, is particularly prominent in the eastern region of Asia [6]. This species is not only famous for its striking white flowers (Fig. 1), but also widely used in urban greening due to its strong adaptability and easy cultivation [7].

As an androdioecy plant, individuals of *C. retusus* are categorized into male and female based on the presence of a pistil in the flowers [8]. Male individuals produce solely male flowers with stamens, while female individuals bloom bisexual flowers featuring both stamens and pistils [6]. These two individuals coexist in the distribution area and can produce offspring through female self-pollination or hybridization [9]. This is a very rare sex system in flowering plants, which has only been clearly reported in a few species, such as *Datisca glomerata* [10], *Tapiscia sinensis* [11], and *Osmanthus delavayi* [12]. This sex system is considered by some studies to be a transitional state of evolution to dioecious plants [13, 14].

The *C. retusus* is widely distributed in China, mainly in the eastern and central southern regions. In addition, it is also distributed in Japan, South Korea and other places [4, 15]. The *C. retusus* exhibits rich morphological variation within the species, especially in the forms of flowers [6]. There are differences in the size, shape, and structure of petals and sepals of its flowers [8]. These morphological variations not only have adaptive significance for their natural survival, but also provide abundant materials for ornamental breeding [6, 8]. The gender differentiation mechanism and morphological variation of *C. retusus* may be related to the role of the *MIKC-type MADS-box* gene family. The *MADS-box* gene family plays a crucial role in regulating flower development and determining floral organ identity [16]. In *C. retusus*, studying this gene family not only provides a deeper understanding of the genetic basis for gender differentiation and morphological variation, but also may provide new molecular tools and strategies for ornamental cultivation, further promoting genetic improvement and variety innovation in *C. retusus* and other ornamental plants.

*MADS-box* gene family encodes a class of transcription factors that control various aspects of growth and development, especially the development of floral organs [17]. *MADS-box* genes are mainly divided into two large groups, termed type I and type II [18, 19]. The type II genes are commonly referred to as *MIKC-type MADS-box* genes, most of these genes are described as controlling the homeotic functions of floral organ [20–22]. The MIKC-type proteins are composed of four typical domains, MADS-box, Intervening, Keratin-like and C-terminal domain [23]. The MADS domain is highly conserved between species and has a dual function: it binds to specific DNA sequences and promotes dimerization [24]. The intervening (I) and keratin-like (K) domains exhibit moderate conservation, which is crucial for the assembly of protein complexes [23, 25, 26]. Meanwhile, the C-terminal domain, known for its diversity, plays a crucial role in the transcriptional activation of MIKC-type MADS-box proteins [23]. In the widely accepted ABCDE model, sepal development is controlled by Class A genes, petal development is controlled by Class A and B genes, stamen development is controlled by Class B and C genes, carpel development is controlled by Class C genes, and Class E genes are involved in the formation of all floral organs [20]. All ABCDE genes belong to the *MIKC-type MADS-box* gene family, except for *APETALA 2* [20]. The *MIKC-type MADS-box* genes is mainly divided into twelve main subfamilies, including AG, SVP, FLC, AP3/PI, ALG12, SOC1, SEP, AGL6, AP1, AGL17, AGL15, MIKC* [17]. AG subfamily for determining the identity of stamen and carpel, as well as the DEF and GLO subfamilies for determining the identity of stamen and petal [17]. Different combinations of *MICK-type MADS-box* genes control the identification of different floral organs [19]. In *Arabidopsis*, the functional deficiency of the *SEPs* gene leads to the transformation of floral organs into leaf-like organs [21]. In rice, the dual mutants of *mads3-mads58* can lead to a complete loss of reproductive organ characteristics of flowers [27]. The *MIKC-type MADS-box* genes controls the formation of floral organs in a complex way of interaction [28].

Due to the important role of the *MIKC-type MADS-box* genes in floral organ development, and our understanding of its characteristics in *C. retusus* is still limited, therefore, in this study, we identified the *MIKC-type MADS-box* genes of *C. retusus* using the latest reference genome information (2n = 2x = 46). *Syringa oblata* [29] and *Olea europaea* [30], like the *C. retusus*, belong to the family Oleaceae and have well assembled genomic information, we also performed genomic collinearity analysis on their *MADS-box* genes. In addition, we also conducted gene structure, conserved motifs and domains, and cis-acting element analysis on the gene and protein sequences of *C. retusus MIKC-type MADS-box* genes. Intended to expand our understanding of the *MADS-box* gene in *C. retusus* through the above analysis. Finally, to understand the role of *MADS-box* genes in gender differentiation of *C. retusus*, transcriptome analysis was performed on the expression patterns of the *MICK-type MADS-box* genes at different development stages of flowers from ‘XueZaoHua’, ‘XueDengLong’, and ‘XueXuan’ varieties, and the results were validated using qRT-PCR in eight varieties of different genders. Our research findings extend our understanding of the *MIKC-type MADS-box* genes in woody plant *C. retusus* and provide a reference for future flower directed breeding of *C. retusus*.

## Result

### Identification and phylogenetic analysis of *MIKC-type CrMADS* genes

By determining the MADS-box domain and K-box domain, combined with the phylogenetic tree, we obtained a total of 61 confidence *MIKC-type CrMADS* genes, and named *CrMADS1*-*CrMADS61* based on its position on the chromosome (Table 1). The amino acid lengths of all MIKC-type CrMADS proteins were 164 – 586 residues, the MWs ranging from 18.97 – 65.92 kDa, the pI values varying from 5.28 – 9.77 (Table 1).

**Table 1:** ID and physicochemical properties of the MIKC type *MADS* gene

ID	name	MW	PI	II	GRAVY
evm.model.Chr11.242.1	MADS25	28,402.13	9.25	72.63	-0.912
evm.model.Chr1.238	MADS2	29,718.72	9.4	60.05	-0.87
evm.model.Chr23.829	MADS61	26,972.55	9.35	62.78	-0.864
evm.model.Chr3.2137	MADS12	25,067.66	7.16	53.11	-0.837
evm.model.Chr16.1573	MADS48	18,970.71	8.8	67.19	-0.831
evm.model.Chr23.112	MADS60	27,145.99	9.53	49.17	-0.821
evm.model.Chr20.1239	MADS55	28,769.47	8.57	72.43	-0.82
evm.model.Chr1.480	MADS3	27,230.07	9.04	47.59	-0.808
evm.model.Chr12.365	MADS31	24,837.23	6	48.76	-0.801
evm.model.Chr11.780	MADS28	25,949.38	5.28	58.65	-0.793
evm.model.Chr21.547	MADS57	24,953.49	8.63	59.63	-0.791
evm.model.Chr3.1435	MADS10	29,438.68	9.01	54.97	-0.76
evm.model.Chr1.2016	MADS6	28,202.32	9.51	62.12	-0.76
evm.model.Chr14.1500	MADS43	25,983.64	9.47	47.16	-0.758
evm.model.Chr18.70	MADS50	28,155.05	8.56	57.84	-0.755
evm.model.Chr7.2242	MADS18	27,650.38	8.3	54.2	-0.754
evm.model.Chr1.237	MADS1	28,487.21	7.66	41.57	-0.745
evm.model.Chr10.1820	MADS22	65,924.95	7.23	43.62	-0.744
evm.model.Chr13.245	MADS35	27,365.47	9.7	54.96	-0.742
evm.model.Chr9.1528	MADS21	27,289.28	9.39	51.31	-0.732
evm.model.Chr18.731.2	MADS51	26,736.49	8.91	47.2	-0.731
evm.model.Chr14.1873	MADS45	25,856.64	9.47	57.71	-0.727
evm.model.Chr14.1748	MADS44	31,188.61	7.93	58.5	-0.72
evm.model.Chr1.752	MADS4	26,113.7	5.57	65.12	-0.715
evm.model.Chr2.896	MADS7	28,088.08	7.58	67.83	-0.714
evm.model.Chr13.1435	MADS40	24,709.27	9.55	53	-0.712
evm.model.Chr13.972	MADS38	24,970.52	9.2	63.94	-0.706
evm.model.Chr11.494	MADS26	27,088.98	9.14	34.62	-0.699
evm.model.Chr16.1576	MADS49	30,471.91	8.82	61.39	-0.698
evm.model.Chr3.109	MADS9	27,286.29	9.5	46.99	-0.696
evm.model.Chr20.579	MADS53	24,748.2	8.83	59.63	-0.694
evm.model.Chr11.240	MADS24	28,241.86	7.65	41.25	-0.692
evm.model.Chr11.1655	MADS29	43,958.46	5.3	47.48	-0.689
evm.model.Chr12.1129	MADS33	27,796.92	8.42	56.37	-0.689
evm.model.Chr19.654	MADS52	26,663.63	9.57	35.58	-0.686
evm.model.Chr10.1821	MADS23	27,658.53	8.39	60.4	-0.685
evm.model.Chr4.1383	MADS16	24,403.12	9.03	50.18	-0.66
evm.model.Chr4.1058	MADS15	28,134.92	8.74	48.93	-0.659
evm.model.Chr13.244	MADS34	27,177.35	9.77	54.75	-0.658
evm.model.Chr1.1693	MADS5	43,657.48	5.88	51.45	-0.639
evm.model.Chr2.1043	MADS8	27,457.99	6.21	58.81	-0.634
evm.model.Chr21.868	MADS58	26,653.49	9.5	49.06	-0.624
evm.model.Chr13.735	MADS37	28,328.36	6.79	54.05	-0.62
evm.model.Chr13.464.1	MADS36	28,913.04	8.76	40.25	-0.613
evm.model.Chr11.712	MADS27	36,529.99	5.65	56.52	-0.571
evm.model.Chr20.902	MADS54	26,612.52	9.39	51.34	-0.565
evm.model.Chr14.535	MADS41	27,169.24	9.4	48.42	-0.541
evm.model.Chr4.613	MADS14	27,235.52	9.66	47.67	-0.529
evm.model.Chr13.1132	MADS39	27,664.89	9.11	47.73	-0.529
evm.model.Chr12.811	MADS32	23,177.67	8.88	36.23	-0.528
evm.model.Chr16.493	MADS47	34,343.15	6.25	54.67	-0.519
evm.model.Chr8.98	MADS19	28,152.84	6.46	53.98	-0.517
evm.model.Chr12.84	MADS30	23,582.18	8.78	56.6	-0.498
evm.model.Chr4.2107	MADS17	23,859.18	8.39	56.85	-0.471
evm.model.Chr14.562	MADS42	32,536.05	5.56	51.3	-0.455
evm.model.Chr3.1888	MADS11	23,703.47	8.81	50.24	-0.454
evm.model.Chr3.2526	MADS13	24,038.93	8.26	46.22	-0.443
evm.model.Chr20.1501	MADS56	27,191.29	8.82	31.85	-0.433
evm.model.Chr21.1475	MADS59	22,696.31	8.58	45.41	-0.402
evm.model.Chr9.784	MADS20	26,174.25	8.27	44.61	-0.371
evm.model.Chr14.1876	MADS46	24,085.23	8.5	39.53	-0.334

**Fig. 2:**
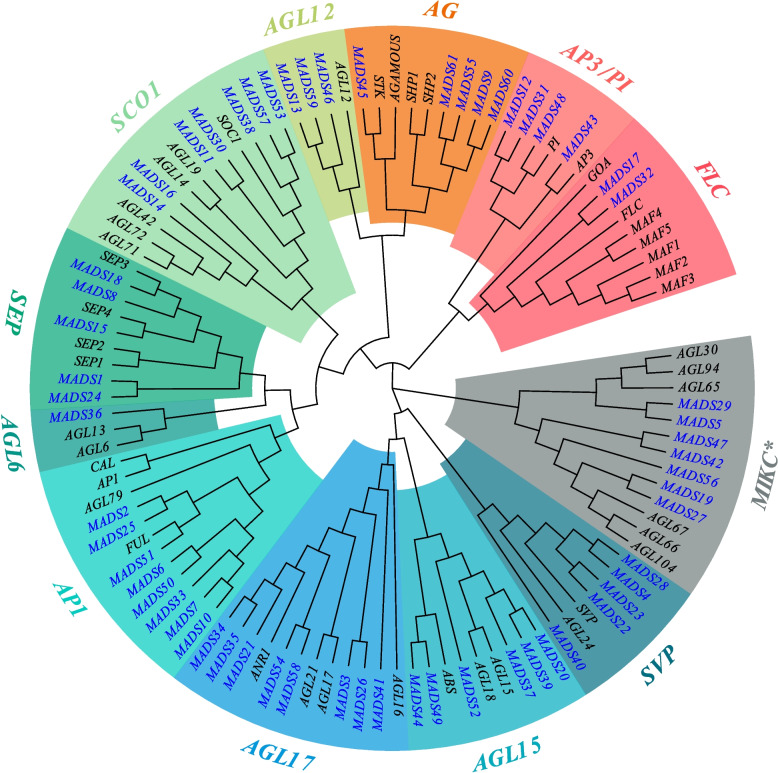
Phylogenetic Tree of *MADS* genes in *C. retusus* and *Arabidopsis*. The blue font represents the *MADS* genes of *C. retusus*, the black font represents the *MADS* genes of *Arabidopsis*, and different colored backgrounds represent different subfamilies

To determine the subfamily classification of 61 *CrMADS*, we constructed a phylogenetic tree by combining their full-length amino acid sequences with the amino acid sequences of 41 type II *MADS-box genes* from *Arabidopsis*. According to phylogenetic analysis, the *CrMADSs* of *C. retusus* were well mapped to 12 subfamilies of *Arabidopsis*, each subfamily containing at least one *CrMADS* (Fig. 2). In 10 subfamilies of all, the number of *CrMADSs* in *C. retusus* increased compared to *Arabidopsis*, namely AP3/PI, AG, AGL12, SOC1, SEP, AP1, AGL17, AGL15, SVP, MIKC*. In the SVP and AGL12 subfamilies, the number of *C. retusus MADS-box* is 3 and 2.5 times that of *Arabidopsis*. However, compared to *Arabidopsis*, the number of *MADS-box* genes in the two subfamilies of *C. retusus* is lower. In the FLC subfamily, *Arabidopsis* has 7 *MADS-box* genes, while *C. retusus* only has two. In the AGL6 subfamily, *Arabidopsis* has two members, while *C. retusus* has only one. This may be due to the genome duplication of Brassicales relative to Oleaceae [31]. The expansion and contraction between different subfamilies may suggest different evolutionary directions between the *C. retusus* and *Arabidopsis*.

### Chromosomal location, genome synteny, and gene duplication of *CrMADSs*

**Fig. 3:**
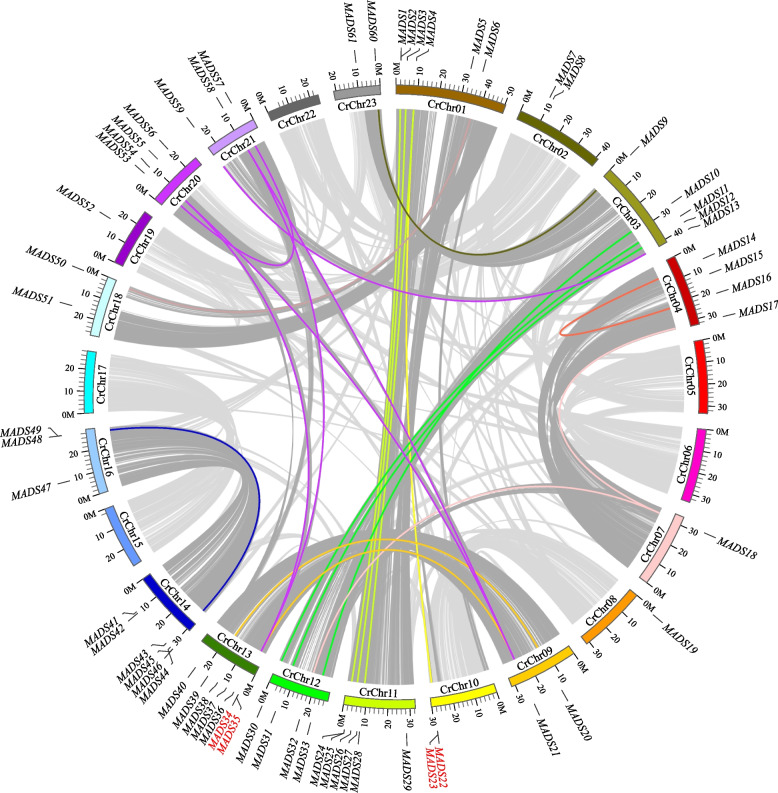
Chromosome distribution and duplication event analysis of *CrMADSs*. Red font represents tandem repeat genes

The *C. retusus* genome consists of 23 chromosomes, with 61 *CrMADS* genes located on 18 of them (Fig. 3). The chromosome 13 have the highest number of *CrMADS* genes, followed by chromosome 1, 11, and 14, which have 6 *CrMADS* genes each. In order to further understand the duplication and evolution of the *C. retusus MADS* genes, we conducted a genomic collinearity analysis of *C. retusus*. Based on the collinearity analysis results, there are extensive collinear segments between the chromosomes of *C. retusus* (Fig. 3). For example, there are collinear segments between chromosome 14 and 16, as well as chromosome 15 and 17. This indicates that the *C. retusus* genome has undergone duplication event. To further investigate the impact of chromosome duplication on the number of *CrMADS* genes, we mapped them to the collinear segments of the chromosome. A total of 21 *CrMADS* gene pairs were identified in the collinearity segments. Some of these genes share collinearity with multiple genes, so these 21 collinear gene pairs involve a total of 34 *CrMADS* genes. This quantity accounts for half of the total *MADS* genes in the *C. retusus* (Fig. 3). This indicates that after genome duplication, these 34 genes underwent retention and evolution, and these genes were preserved within the collinear segments. In addition, we also identified tandem duplication *CrMADS* genes in the *C. retusus* genome. There are a total of 4 tandem duplication genes, namely *CrMADS22* and *CrMADS23*, as well as *CrMADS34* and *CrMADS35* (Fig. 3). Therefore, segmental duplication and tandem duplication both contribute to the amplification and evolution of the *CrMADS* genes, increasing the diversity of the *C. retusus MADS* gene family. At the same time, this also explains to some extent the quantitative expansion of some subfamilies relative to *Arabidopsis*. For example, the *CrMADS9* and *CrMADS60* genes in the AG subfamily are located on chromosome 3 and 23, respectively. The corresponding segments of these two chromosomes have a clear collinearity relationship, and these two genes are located within them. This indicates that these two genes may have been fixed into two by the same gene after undergoing possible genome duplication. The same examples also include *CrMADS12* and *CrMADS31* in the AP3/PI subfamily, as well as *CrMADS13* and *CrMADS59* in the AGL12 subfamily. In addition, the tandem repeats between *CrMADS22* and *CrMADS23*, as well as the tandem repeats between *CrMADS34* and *CrMADS35*, to some extent explain the increase in the number of members in the SVP and AGL17 subfamilies.

Conducting inter-species comparative analysis of the *C. retusus* genome, along with the genomes of *S. oblata* and *O. europaea*, would be beneficial for a better understanding of the evolution of the *MADS-box* gene family. *S. oblata*, *O. europaea*, and *C. retusus* all have the same number of chromosomes, with each species having 23 chromosomes. Based on the collinearity analysis results, it can be observed that there is a strong collinearity relationship between the corresponding chromosomes across these species (Fig. S1). However, there have been certain chromosome segment rearrangements that have occurred between these species. We have identified *MADS-box* genes located within these collinear regions, and we have found a total of 79 pairs of *MADS-box* genes located within the collinear regions between *C. retusus* and *O. europaea* (Fig. S1, Table 2). Between *C. retusus* and *S. oblata*, this number is 64 pairs. It is suggested that *C. retusus* has a closer genetic relationship with *O. europaea*. Subsequently, the *Ka/Ks* values were calculated for all collinear *MADS-box* gene pairs, and the results showed that the *Ka/Ks* values for all *MADS-box* gene pairs were less than 1 (Table 3). Therefore, after the divergence of *C. retusus* and these two species, the *MADS-box* genes have undergone purifying selection. The above results indicate that *MADS-box* genes in the Oleaceae family are highly conserved.

**Table 2:** The *MADS-box* genes sharing relationship between C. retusus, *S. oblata*, and *O. europaea*

OeMADS	CrMADS	SoMADS
EVM0044384.1	MADS1	Ob0203822.1
EVM0003838.1	MADS1	Ob0203822.1
EVM0055724.1	MADS10	Ob0241012.1
EVM0046369.1	MADS10	Ob0241012.1
EVM0050142.1	MADS11	Ob0206343.1
EVM0031933.1	MADS12	Ob0206588.1
EVM0018088.1	MADS12	Ob0206588.1
EVM0047689.1	MADS13	
EVM0045916.1	MADS14	Ob0235820.1
EVM0047368.1	MADS15	
EVM0055112.1	MADS16	Ob0235060.1
EVM0001946.1	MADS18	Ob0229475.1
EVM0004662.1	MADS2	Ob0203821.1
EVM0034888.1	MADS2	Ob0203821.1
EVM0005398.1	MADS20	
EVM0044201.1	MADS20	
EVM0019358.1	MADS21	
EVM0057982.1	MADS23	
EVM0003838.1	MADS24	Ob0203822.1
EVM0044384.1	MADS24	Ob0203822.1
EVM0034888.1	MADS25	Ob0203821.1
EVM0004662.1	MADS25	Ob0203821.1
EVM0056648.1	MADS26	Ob0222843.1
EVM0030136.1	MADS28	Ob0223189.1
EVM0040770.1	MADS28	Ob0223189.1
EVM0015055.1	MADS29	Ob0224274.1
EVM0029370.1	MADS29	Ob0224274.1
EVM0028291.1	MADS3	Ob0203514.1
EVM0050142.1	MADS30	Ob0206343.1
EVM0018088.1	MADS31	Ob0206588.1
EVM0031933.1	MADS31	Ob0206588.1
EVM0051245.1	MADS32	
EVM0055724.1	MADS33	Ob0241012.1
EVM0046369.1	MADS33	Ob0241012.1
EVM0019358.1	MADS34	
EVM0008480.1	MADS36	
EVM0041296.1	MADS37	Ob0232861.1
EVM0007323.1	MADS37	Ob0232861.1
EVM0028307.1	MADS38	Ob0225922.1
EVM0044201.1	MADS39	Ob0226159.1
EVM0005398.1	MADS39	Ob0226159.1
EVM0040770.1	MADS4	Ob0223189.1
EVM0030136.1	MADS4	Ob0223189.1
EVM0043694.2	MADS40	Ob0226579.1
EVM0059472.1	MADS41	
EVM0011280.1	MADS41	
EVM0010572.1	MADS42	Ob0239951.1
EVM0052147.1	MADS42	Ob0239951.1
EVM0030377.1	MADS43	Ob0217127.1
EVM0014143.1	MADS44	Ob0238724.1
EVM0045779.2	MADS44	Ob0238724.1
EVM0047006.1	MADS45	Ob0217663.1
EVM0053307.1	MADS46	Ob0238582.1
EVM0052147.1	MADS47	Ob0239951.1
EVM0010572.1	MADS47	Ob0239951.1
EVM0003784.1	MADS48	
EVM0014143.1	MADS49	Ob0238724.1
EVM0045779.2	MADS49	Ob0238724.1
EVM0015055.1	MADS5	Ob0201990.1
EVM0029370.1	MADS5	Ob0201990.1
EVM0055226.1	MADS50	
EVM0007259.1	MADS51	Ob0200958.1
EVM0008699.1	MADS52	Ob0218806.1
EVM0019358.1	MADS54	Ob0251971.1
EVM0012737.1	MADS54	Ob0251971.1
EVM0015960.1	MADS54	Ob0251971.1
EVM0041998.1	MADS55	Ob0253150.1
EVM0022767.1	MADS55	Ob0253150.1
EVM0052524.1	MADS56	Ob0250363.1
EVM0053426.1	MADS59	
EVM0043114.1	MADS6	Ob0201611.1
EVM0055226.1	MADS6	Ob0201611.1
EVM0042985.1	MADS60	Ob0252294.1
EVM0022767.1	MADS61	Ob0253150.1
EVM0041998.1	MADS61	Ob0253150.1
EVM0049448.1	MADS7	Ob0247305.1
EVM0057738.1	MADS8	Ob0210902.1
EVM0042985.1	MADS9	Ob0204431.1
EVM0030144.1	MADS9	Ob0204431.1

**Table 3:** The *Ka*, *Ks*, and *Ka*/*Ks* values between collinear *MADS* gene pairs

Seq_1	Seq_2	Ka	Ks	Ka_Ks
EVM0047006.1	evm.model.Chr14.1873	0.00579	0.105934	0.054654
EVM0001946.1	evm.model.Chr7.2242	0.012547	0.114251	0.10982
EVM0030377.1	evm.model.Chr14.1500	0.015437	0.118987	0.129737
EVM0022767.1	evm.model.Chr23.829	0.021615	0.155322	0.13916
EVM0055724.1	evm.model.Chr12.1129	0.017847	0.125291	0.142448
EVM0057738.1	evm.model.Chr2.1043	0.021936	0.139551	0.15719
EVM0050142.1	evm.model.Chr3.1888	0.021025	0.125291	0.167806
EVM0004662.1	evm.model.Chr1.238	0.062412	0.357496	0.174581
EVM0041998.1	evm.model.Chr23.829	0.048844	0.275974	0.176989
EVM0018088.1	evm.model.Chr12.365	0.049049	0.267429	0.18341
EVM0003838.1	evm.model.Chr11.240	0.040356	0.219805	0.183601
EVM0041998.1	evm.model.Chr20.1239	0.019706	0.107026	0.184119
EVM0034888.1	evm.model.Chr11.242.1	0.064792	0.347289	0.186564
EVM0044384.1	evm.model.Chr1.237	0.040387	0.211125	0.191292
EVM0040770.1	evm.model.Chr1.752	0.055723	0.28355	0.196521
EVM0004662.1	evm.model.Chr11.242.1	0.021475	0.10793	0.198969
EVM0030136.1	evm.model.Chr11.780	0.045337	0.220702	0.205421
EVM0031933.1	evm.model.Chr3.2137	0.052195	0.247505	0.210884
EVM0022767.1	evm.model.Chr20.1239	0.062661	0.293845	0.213246
EVM0003838.1	evm.model.Chr1.237	0.019313	0.083733	0.230655
EVM0042985.1	evm.model.Chr23.112	0.031563	0.136581	0.231097
EVM0040770.1	evm.model.Chr11.780	0.020802	0.089771	0.231722
EVM0055724.1	evm.model.Chr3.1435	0.052139	0.224562	0.232182
EVM0044384.1	evm.model.Chr11.240	0.019522	0.082101	0.23778
EVM0014143.1	evm.model.Chr16.1576	0.064307	0.269775	0.238371
EVM0030136.1	evm.model.Chr1.752	0.021686	0.09091	0.238543
EVM0008480.1	evm.model.Chr13.464.1	0.023122	0.093159	0.2482
EVM0046369.1	evm.model.Chr12.1129	0.049265	0.195697	0.251742
EVM0045779.2	evm.model.Chr16.1576	0.043382	0.17199	0.252233
EVM0014143.1	evm.model.Chr14.1748	0.021418	0.083598	0.256205
EVM0056648.1	evm.model.Chr11.494	0.03048	0.116774	0.261014
EVM0028291.1	evm.model.Chr1.480	0.027798	0.100149	0.277569
EVM0015055.1	evm.model.Chr11.1655	0.028404	0.099057	0.286748
EVM0003784.1	evm.model.Chr16.1573	0.050987	0.177513	0.28723
EVM0019358.1	evm.model.Chr20.902	0.147954	0.496101	0.298234
EVM0042985.1	evm.model.Chr3.109	0.068188	0.224336	0.303955
EVM0045779.2	evm.model.Chr14.1748	0.078169	0.246963	0.316522
EVM0049448.1	evm.model.Chr2.896	0.037772	0.117821	0.320589
EVM0047368.1	evm.model.Chr4.1058	0.049152	0.151211	0.325058
EVM0043114.1	evm.model.Chr1.2016	0.082773	0.244712	0.338249
EVM0047689.1	evm.model.Chr3.2526	0.043732	0.128312	0.340826
EVM0015055.1	evm.model.Chr1.1693	0.086006	0.245922	0.349728
EVM0059472.1	evm.model.Chr14.535	0.072481	0.20664	0.35076
EVM0053307.1	evm.model.Chr14.1876	0.054134	0.154168	0.351135
EVM0011280.1	evm.model.Chr14.535	0.036642	0.103754	0.353167
EVM0044201.1	evm.model.Chr13.1132	0.130854	0.368906	0.354709
EVM0012737.1	evm.model.Chr20.902	0.064992	0.18306	0.355032
EVM0019358.1	evm.model.Chr9.1528	0.033231	0.091287	0.364028
EVM0055226.1	evm.model.Chr1.2016	0.068364	0.185613	0.368314
EVM0005398.1	evm.model.Chr9.784	0.118052	0.316517	0.372973
EVM0052524.1	evm.model.Chr20.1501	0.094094	0.24763	0.379979
EVM0034888.1	evm.model.Chr1.238	0.040977	0.103056	0.397624
EVM0050142.1	evm.model.Chr12.84	0.089132	0.219817	0.405483
EVM0052147.1	evm.model.Chr16.493	0.134849	0.329778	0.408908
EVM0051245.1	evm.model.Chr12.811	0.059789	0.144538	0.413659
EVM0010572.1	evm.model.Chr14.562	0.133693	0.321703	0.415577
EVM0043694.2	evm.model.Chr13.1435	0.055532	0.133505	0.415956
EVM0057982.1	evm.model.Chr10.1821	0.089539	0.214165	0.418085
EVM0030144.1	evm.model.Chr3.109	0.0486	0.115744	0.41989
EVM0031933.1	evm.model.Chr12.365	0.035816	0.085077	0.420982
EVM0041296.1	evm.model.Chr13.735	0.158795	0.368547	0.430868
EVM0044201.1	evm.model.Chr9.784	0.058603	0.130991	0.447384
EVM0046369.1	evm.model.Chr3.1435	0.031362	0.069014	0.454427
EVM0007323.1	evm.model.Chr13.735	0.048527	0.10289	0.471642
EVM0045916.1	evm.model.Chr4.613	0.167671	0.344163	0.487184
EVM0055112.1	evm.model.Chr4.1383	0.04518	0.088873	0.50837
EVM0028307.1	evm.model.Chr13.972	0.050834	0.098967	0.513644
EVM0029370.1	evm.model.Chr11.1655	0.17827	0.346968	0.513794
EVM0053426.1	evm.model.Chr21.1475	0.055253	0.106737	0.517657
EVM0007259.1	evm.model.Chr18.731.2	0.064401	0.123956	0.519544
EVM0008699.1	evm.model.Chr19.654	0.073094	0.139473	0.524072
EVM0055226.1	evm.model.Chr18.70	0.054037	0.09719	0.555995
EVM0010572.1	evm.model.Chr16.493	0.077243	0.138816	0.556447
EVM0018088.1	evm.model.Chr3.2137	0.043467	0.078112	0.556476
EVM0015960.1	evm.model.Chr20.902	0.043517	0.073917	0.588734
EVM0029370.1	evm.model.Chr1.1693	0.153916	0.258123	0.596289
EVM0005398.1	evm.model.Chr13.1132	0.052279	0.077947	0.670706
EVM0019358.1	evm.model.Chr13.244	0.06577	0.092644	0.709917
EVM0052147.1	evm.model.Chr14.562	0.091616	0.094679	0.967655
Ob0217663.1	evm.model.Chr14.1873	0.009679	0.185556	0.052163
Ob0203822.1	evm.model.Chr11.240	0.03662	0.280709	0.130455
Ob0217127.1	evm.model.Chr14.1500	0.021357	0.158984	0.134336
Ob0253150.1	evm.model.Chr23.829	0.024554	0.181826	0.135039
Ob0203821.1	evm.model.Chr11.242.1	0.064474	0.470427	0.137055
Ob0203822.1	evm.model.Chr1.237	0.021159	0.148969	0.142037
Ob0253150.1	evm.model.Chr20.1239	0.047562	0.290702	0.163612
Ob0222567.1	evm.model.Chr1.237	0.03843	0.221753	0.1733
Ob0241012.1	evm.model.Chr3.1435	0.042823	0.239679	0.178669
Ob0210902.1	evm.model.Chr2.1043	0.03129	0.172284	0.181619
Ob0222567.1	evm.model.Chr11.240	0.030307	0.160971	0.188277
Ob0241012.1	evm.model.Chr12.1129	0.026993	0.139369	0.193683
Ob0238724.1	evm.model.Chr16.1576	0.058897	0.270368	0.217841
Ob0247305.1	evm.model.Chr2.896	0.056447	0.249704	0.226055
Ob0252294.1	evm.model.Chr23.112	0.04758	0.203615	0.233676
Ob0238694.1	evm.model.Chr16.1576	0.050319	0.209522	0.240162
Ob0223189.1	evm.model.Chr11.780	0.034628	0.138337	0.250314
Ob0231820.1	evm.model.Chr10.1820	0.057049	0.226551	0.251814
Ob0251971.1	evm.model.Chr21.868	0.044908	0.174168	0.257844
Ob0224274.1	evm.model.Chr11.1655	0.060463	0.22843	0.264689
Ob0201990.1	evm.model.Chr11.1655	0.075093	0.281314	0.266936
Ob0204431.1	evm.model.Chr3.109	0.042715	0.15967	0.267522
Ob0251570.1	evm.model.Chr21.547	0.068521	0.255977	0.267685
Ob0200958.1	evm.model.Chr18.731.2	0.090226	0.323508	0.2789
Ob0204431.1	evm.model.Chr23.112	0.072063	0.25458	0.283067
Ob0203821.1	evm.model.Chr1.238	0.064792	0.224701	0.288347
Ob0238582.1	evm.model.Chr14.1876	0.046951	0.161941	0.28993
Ob0245459.1	evm.model.Chr18.731.2	0.087714	0.299761	0.292614
Ob0203154.1	evm.model.Chr11.780	0.068099	0.228651	0.29783
Ob0238724.1	evm.model.Chr14.1748	0.078435	0.257148	0.305019
Ob0243927.1	evm.model.Chr20.579	0.050224	0.162045	0.309937
Ob0238694.1	evm.model.Chr14.1748	0.077799	0.248023	0.313675
Ob0223189.1	evm.model.Chr1.752	0.078157	0.244808	0.319258
Ob0251570.1	evm.model.Chr20.579	0.048755	0.150006	0.325017
Ob0201611.1	evm.model.Chr1.2016	0.049308	0.144729	0.340694
Ob0218806.1	evm.model.Chr19.654	0.121765	0.353304	0.344648
Ob0222571.1	evm.model.Chr1.238	0.229734	0.665141	0.345391
Ob0201990.1	evm.model.Chr1.1693	0.076805	0.217849	0.352561
Ob0235060.1	evm.model.Chr4.1383	0.059131	0.157646	0.375089
Ob0243927.1	evm.model.Chr21.547	0.052195	0.13755	0.379463
Ob0203514.1	evm.model.Chr1.480	0.024343	0.060992	0.39912
Ob0226159.1	evm.model.Chr13.1132	0.0818	0.199226	0.410587
Ob0239951.1	evm.model.Chr14.562	0.133764	0.308735	0.433265
Ob0206588.1	evm.model.Chr12.365	0.193171	0.434961	0.444111
Ob0206343.1	evm.model.Chr12.84	0.132253	0.292187	0.452631
Ob0222843.1	evm.model.Chr11.494	0.101365	0.223517	0.453499
Ob0205794.1	evm.model.Chr12.1129	0.184334	0.402028	0.458511
Ob0252294.1	evm.model.Chr3.109	0.10011	0.216784	0.461795
Ob0232861.1	evm.model.Chr13.735	0.099948	0.215205	0.464432
Ob0206588.1	evm.model.Chr3.2137	0.104481	0.216898	0.481703
Ob0229475.1	evm.model.Chr7.2242	0.17606	0.361853	0.486551
Ob0205794.1	evm.model.Chr3.1435	0.198172	0.403602	0.491009
Ob0239951.1	evm.model.Chr16.493	0.118751	0.235931	0.50333
Ob0251971.1	evm.model.Chr20.902	0.042796	0.083371	0.513317
Ob0206343.1	evm.model.Chr3.1888	0.083953	0.160018	0.524648
Ob0217666.1	evm.model.Chr14.1876	0.133279	0.236486	0.563581
Ob0203154.1	evm.model.Chr1.752	0.072956	0.123799	0.589308
Ob0225922.1	evm.model.Chr13.972	0.084111	0.139767	0.601792
Ob0226579.1	evm.model.Chr13.1435	0.108009	0.173097	0.623977
Ob0203550.1	evm.model.Chr1.480	0.029278	0.043208	0.677597
Ob0225601.1	evm.model.Chr13.735	0.062136	0.090141	0.689324
Ob0250363.1	evm.model.Chr20.1501	0.104816	0.150064	0.698474
Ob0235820.1	evm.model.Chr4.613	0.188864	0.268752	0.702744
Ob0242400.1	evm.model.Chr12.84	0.081248	0.114094	0.712117

In order to determine the phylogenetic relationship between the *MADS-box* genes of these three species in the Oleaceae family, we constructed a phylogenetic tree by combining the *MADS-box* genes of *O. europaea* and *S. oblata* with those of *C. retusus*. Interestingly, even within the same family, the *MADS-box* gene undergoes a certain degree of differentiation (Fig. S3). For example, in the AGL17 subfamily, the *MADS-box* gene of *S. oblata* is almost in a different evolutionary branch from that of *C. retusus* and *O. europaea*, while in the AGL15 subfamily, both *C. retusus* and *O. europaea* have two members, while *S. oblata* only has one. However, overall, the corresponding relationship between *MADS-box* genes in the *C. retusus*, *O. europaea*, and *S. oblata* is still very good.

### Gene structure, conserved protein motifs and domain analysis

The gene structure of *CrMADS* genes, as shown in the Fig. S2, is characterized by multiple exons and long introns, similar to other species in this regard [32–34]. Most *C. retusus MADS-box* genes have 7 or 8 exons, accounting for 25/61 and 26/61, respectively. At this point, *C. retusus* is similar to other species. Additionally, *CrMADS22* has the highest numbers of exons, with a total of 12. On the other hand, *CrMADS32* and *CrMADS48* have the fewest number of exons, with a total of 6. Overall, the average number of exons of 61 *CrMADS* genes is 7.8. The structure of *MADS-box* genes varies significantly among different subfamilies but remains relatively conserved within each subfamily. In the AP3/PI subfamilies, *CrMADS* genes commonly have shorter introns, while in the AGL17 and FLC subfamilies, *CrMADS* genes generally have longer introns (Fig. S2).

**Fig. 4:**
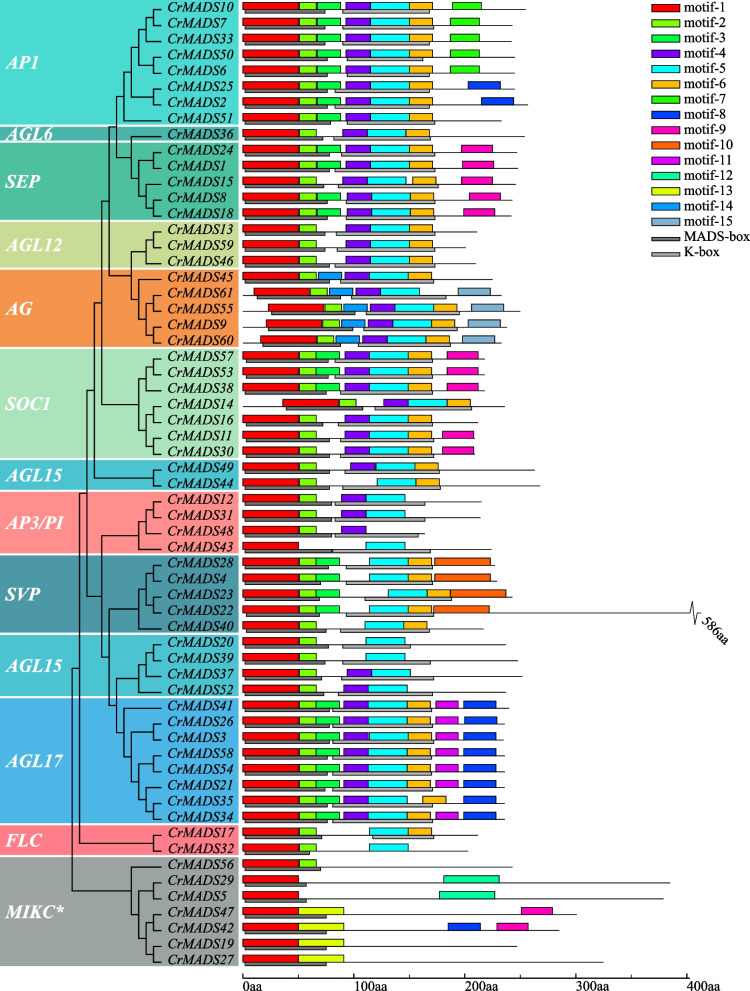
Phylogenetic relationships, domains, and motifs composition of *CrMADSs*

Furthermore, we identified the conserved domains and motifs of 61 CrMADS protein sequences. The results revealed that all CrMADS proteins possess the MADS-box domain, corresponding to motif-1 (Fig. 4). Additionally, the K-box domain is highly conserved in non-MIKC* subfamilies, corresponding to motif-4, motif-5 and motif-6. However, typical K-box domains and their corresponding motifs were not identified in the MIKC* subfamily. This could be due to the rearrangement of exons in ancient MIKC* genes [18, 35]. In addition to highly conserved motifs, there are also some motifs that only exist in specific subfamilies. For example, the AP1 subfamily possesses a unique motif, motif-7. Similarly, the AG subfamily has motif-14 and motif-15 as its distinctive motifs. These subfamily-specific motifs are often found at the C-terminus of CrMADS protein sequences, indicating that the C-terminus of CrMADS proteins possesses a relatively higher structural diversity. The conserved MADS-box and K-box domains, along with the diverse C-terminal domains, confirm the previous view that the CrMADS family proteins have undergone functional differentiation while retaining the conserved *MADS-box* genes shared functions [36–38]. Considering that tassels, as perennial woody plants, may have differences in the distribution of motifs compared to Arabidopsis.

### Identification and enrichment analysis of *cis*-acting elements in promoters of *CrMADSs*

**Fig. 5:**
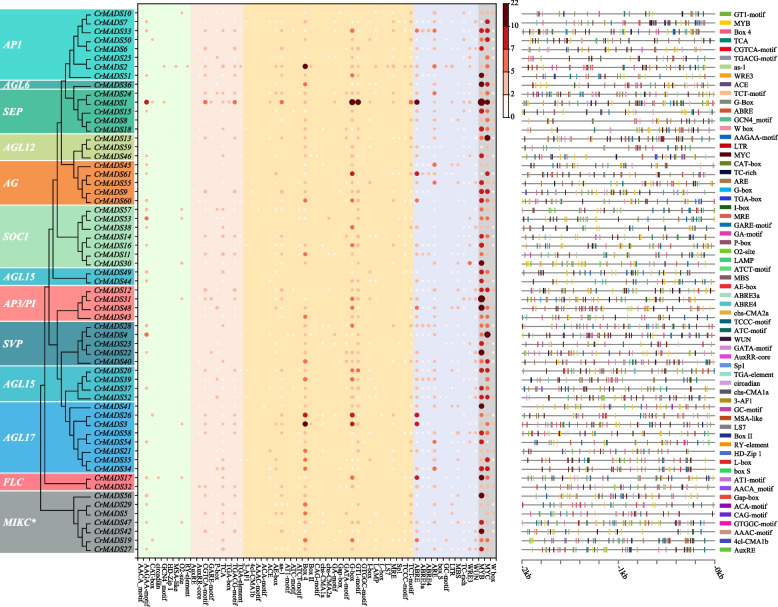
Enrichment and distribution of cis-acting elements in the promoter of *CrMADSs*. The color and size of the point indicate the number of elements, while red and large dimensions indicate more element

In gene regulation, promoter cis-acting elements play crucial roles in controlling gene expression [39]. The promoters of 61 *CrMADS* genes were analyzed using PlantCARE tool [40]. The identified cis-acting elements were than classified based on their potential regulatory functions. In our analysis, we identified a total of 2760 number of specific cis-acting elements in the 2000 bp upstream sequence of the 61 *CrMADS* genes (Fig. 5). According to their functional annotations, they are divided into 5 categories, namely growth-related elements, hormone-responsive elements, light-responsive elements, stress-responsive elements, and transcription factor binding sites. Among them, the largest quantity is the light-responsive elements (879), followed by transcription factor binding sites (784) and stress-responsive elements (607). The least abundant are hormone-responsive elements and growth-related elements (288 and 202, respectively). This suggests that the *CrMADS* genes may respond to light and stress stimuli, and they may be regulated by various transcription factors. Among the 61 *CrMADS* genes, *CrMADS1* possesses the highest number of cis-acting elements. A total of 136 elements were identified in its promoter sequence, which is significantly higher than average count of 45.2 (Fig. 5). These include 22 MYB elements, 14 G-box elements, and 12 GT1-motif. The significantly higher number of cis-acting elements in *CrMADS1* compared to the average indicates that it may be plays an important role in floral organ development.

### The differential expression patterns of the *C. retusus MADS-box* genes in androdioecy flowers

**Fig. 6:**
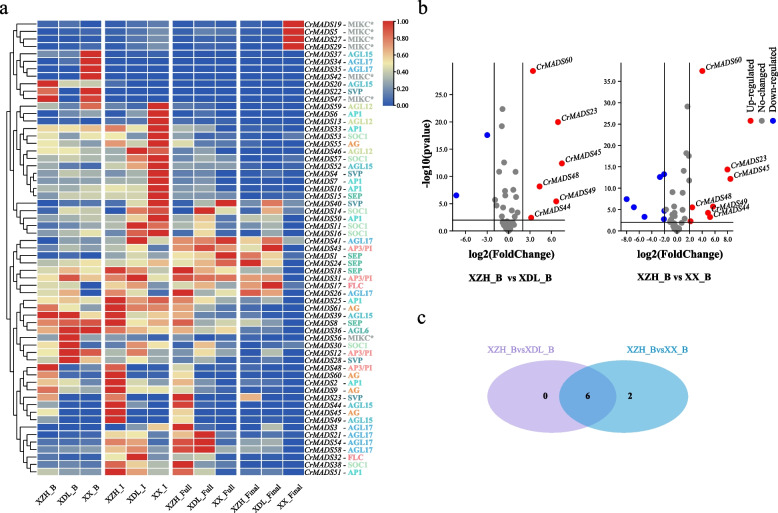
Expression and differential analysis of *CrMADSs*. **a** Heatmap of the expression of 61 *CrMADSs* in flower organs at four stages of three varieties. The expression heatmap of 61 MADS in flower organs of three varieties at four stages. B represents the Bud stage, I represents the Initial flowering stage, Full represents the Full flowering stage, and Final represents the Final flowering stage. **b** Genes differentially expressed in the bud stage between ‘XZH’ and two other varieties. **c** Wayne diagram of differentially expressed genes

The *C. retusus*, as an androdioecy plant, exists in nature only as hermaphroditic flower individuals and male flower individuals. To investigate the expression patterns of *CrMADS* genes during the development of *C. retusus* flowers in different sexes, we selected floral organs from three varieties: ‘XZH’ (hermaphroditic flowers), ‘XDL’ (male flowers), and ‘XX’ (male flowers). We measured their transcriptomes during four development stages: bud stage (B), initial flowering stage (I), full flowering stage (Full), and final flowering stage (Final). The heatmap (Fig. 6) displays the expression patterns of 61 *CrMADS* genes. The 61 *CrMADS* genes exhibit different expression patterns during the flower development process in different varieties. It should be noted that *CrMADS* genes from the same subfamily often exhibit similar expression patterns. For example, within the MIKC* subfamily, *MADS-box* genes such as *CrMADS19*, *CrMADS5*, *CrMADS27*, and *CrMADS29*; and within the SEP subfamily, *CrMADS1*, *CrMADS24*, and *CrMADS18* exhibit similar expression patterns (Fig. 6).

**Fig. 7:**
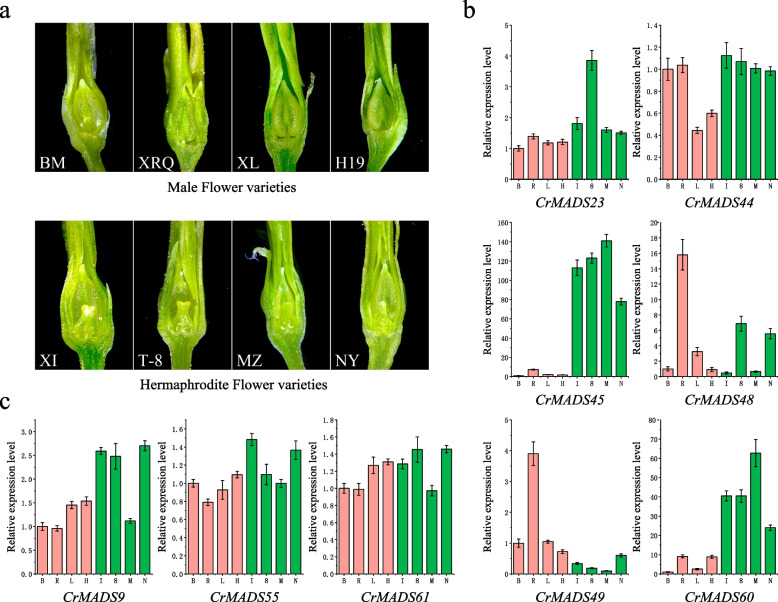
The floral organs of eight varieties and the expression patterns of *CrMADSs* in floral organs. **a** Vertical sectional views of floral organs during the bud stage. Among them, BM, XRQ, XL, H19 are male flower individuals, XI, T-8, MZ, NY are bisexual flower individuals. Both individuals have stamens, while only bisexual individuals have pistils. **b** The relative expression levels of differentially expressed *CrMADSs* in flower organs of 8 varieties. **c** The relative expression levels of *CrMADSs* in the AG subfamily in flower organs of 8 varieties

Compared to the ‘XX’ and ‘XDL’ varieties, ‘XZH’ exhibits specific upregulation of certain genes during the bud stage, initiation flowering, and full flowering stage. For instance, genes such as *CrMADS60* and *CrMADS48* show higher expression levels in ‘XZH’ than in ‘XX’ and ‘XDL’. ‘XX’ and ‘XDL’ are male individuals, while ‘XZH’ is female individuals. In ‘XZH’, there are both complete pistils and stamens, while in ‘XX’ and ‘XDL’, only stamens exist and the pistils are completely absent. Therefore, we assume that *MADS-box* genes related to carpel development, such as class C genes, will exhibit differential expression between ‘XDL’ and the other two varieties. In order to identify genes in the *MADS-box* family that may be involved in gender differentiation of *C. retusus* flower, we screened for differentially upregulated *CrMADS* genes during the bud stage in ‘XZH’ compared to ‘XX’ or ‘XDL’ (Fig. 6). We then took the intersection of these genes, resulting in a total of six differential genes identified as gender-related *CrMADS* genes (Fig. 6). These six differential genes primarily belong to the AP3/PI, SVP, AG, and AGL15 subfamilies. Subsequently, to validate the expression patterns of these six genes in flowers of different genders, we selected an additional eight varieties, including four male flower individuals (‘XL’, ‘BM’, ‘H19’, ‘XRQ’) and four hermaphrodite flower individuals (‘XI’, ‘NY’, ‘T-8’, ‘MZ’) (Fig. 7). The qRT-PCR was performed to test the expression patterns of these six candidate genes in the bud flowers of these eight varieties. The results indicated that out of the six candidate genes, only *CrMADS45* and *CrMADS60* exhibited significant gender-specific expression patterns (Fig. 7). Among them, *CrMADS45* showed expression levels ranging from 77.8 to 141.1 times higher in hermaphrodite flowers compared to male flowers. The expression fold of *CrMADS60* is 23.9 to 62.7 times. Importantly, it is noteworthy that both *CrMADS45* and *CrMADS60* both belong to the AG subfamily, whose members in *Arabidopsis* have been shown to play critical roles in carpel development [41, 42].

In addition to *CrMADS45* and *CrMADS60*, there are three other members in the AG subfamily of *C. retusus*, namely *CrMADS9*, *CrMADS55*, and *CrMADS61* (Fig. 2). To determine whether the three were also involved in the gender differentiation of *C. retusus*, we also measured their expression levels in eight varieties. However, the results showed that *CrMADS9*, *CrMADS55*, and *CrMADS61* did not exhibit the same expression patterns as *CrMADS45* and *CrMADS60* (Fig. 7). Hence, we conclude that only *CrMADS45* and *CrMADS60* play important roles in the differentiation of male flowers and hermaphrodite flowers in *C. retusus*.

## Discussion

As a garden ornamental plant, the cultivation of *C. retusus* focuses on the diversity of flower morphology [6]. The morphology of plant flowers is mainly determined by the arrangement and quantity of floral organs [35]. In *Arabidopsis*, the *MADS-box* gene family consists of 108 members and is divided into two main group: type I and type II [17]. Among them, the type II genes, specifically the *MIKC-type MADS-box* genes, are primarily associated with flower organ development, there are a total of 45 members in the *MIKC-type MADS-box* gene family of *Arabidopsis* [16]. The number of *MADS-box* genes varies significantly among different species. Here, we identified a total of 61 *MIKC-type MADS-box* genes in the genome of *C. retusus*. This number is higher compared to several other dicotyledonous plants, such as *Arabidopsis* with 45 *MIKC-type MADS-box* genes, sacred lotus with 28 genes [43], and sesame with 33 genes [34]. However, the number of *MIKC-type MADS-box* genes in *C. retusus* is significantly lower than that in the monocotyledonous plant wheat, which has 201 *MIKC-type MADS-box* genes [44]. Within the Oleaceae family, such as *S. oblata*, *O. europaea*, and *C. retusus*, the number of *MADS-box* genes is generally similar (Fig. S3). This indicates that *MIKC-type MADS-box* genes have undergone complex expansions and losses during the evolution of different species.

In previous studies, *MIKC-type MADS-box* genes have been classified into 12 major subfamilies based on their functional characteristics and sequence similarity [17]. Here, 61 *MIKC-type MADS-box* genes in *C. retusus* have been successfully mapped to the 12 subfamilies, with each subfamily containing at least one *CrMADS* genes. However, compared to *Arabidopsis*, *C. retusus MADS-box* genes have undergone significant doubled in several subfamilies, such as the AGL12 and SVP subfamilies. In contrast, in some other subfamilies, such as the FLC, the number of *MADS-box* gens in *C. retusus* has significantly halved. Both the FLC and SVP subfamilies have been shown to be involved in the transition to flowering, while AGL12 is considered to be associated with root development and the transition to flowering [45–47]. Therefore, the expansions and contractions of *C. retusus* different *MADS-box* subfamilies reflect its potential adaptations in various aspects of growth and development.

*C. retusus*, as an androdiecious plant, exists in nature as only two types of individuals: male flower individuals that produce only male flowers and hermaphroditic flower individuals that produce only hermaphrodite flowers [6, 9]. In our study, by analyzing the transcriptomes of different gendered varieties of *C. retusus* flowers, we identified 6 genes that showed differential expression during the floral bud stage, include members of the AP3/PI, AG, SVP, and AGL15 subfamilies. Previous studies have shown that AP3/PI predominantly functions in determining the identity of petals and stamens, known as the B-class function [48, 49]. AG primarily acts in determining the identity of carpels and stamens, referred to as the C-class function [27]. SVP and AGL15 play significant roles in the transition to flowering, and AGL15 also plays a role in embryogenesis [45, 50, 51]. In the process of flower development, there may be differences between ‘XZH’ and ‘XX’ /’XDL’ in these aspects. However, in the subsequent qRT-PCR results of eight varieties, only *CrMADS45* and *CrMADS60* showed significant differential expression in male flowers and hermaphroditic flowers. Interestingly, both of these two genes belong to the AG subfamily. Previous studies have shown that members of the AG subfamily mainly play a role in C-class functions, and some members also play D-class functions. C-class genes are associated with the formation of both stamens and pistils [52, 53]. The genes of the AG subfamily should also regulate the development of pistils in *C. retusus* through this very complex mechanism, and thus affect their gender differentiation. Just as, subsequent measurements of the expression levels of the other three members of the AG subfamily did not show the same expression pattern, indicating that not all AG subfamily genes are involved in the gender differentiation of *C. retusus*, or play different roles in this process. By reviewing our results on the cis-acting elements of promoters and conserved motifs of amino acid sequences, we hope to find relevant information on why these genes in the AG subfamily exhibit different performances. Unfortunately, we have not found any useful clues. However, future research on the population of *C. retusus* may provide an answer.

In summary, our study involved the sequence analysis and the expression analysis of the *MADS-box* genes in a recently sequenced species called *C. retusus* (Chinese Fringe tree), and identified two *MADS-box* genes that may be involved in sex differentiation of *C. retusus*. This research provides a valuable reference for future studies on gene function and breeding strategies in *C. retusus*.

## Conclusion

In this study, we identified 61 *MIKC-type MADS-box* genes within the *C. retusus* genome. Through phylogenetic analysis, these genes were classified into 12 distinct subfamilies. The analysis of genomic collinearity reveals the duplication events experienced by *CrMADS* genes and the purification selection accompanying the diversification process of plants in the Oleaceae family. Conservation domain and motif analyses demonstrated their structural conservation. The determination of gene expression levels in flower organs of different genders showed that *CrMADS45* and *CrMADS60* exhibited significant high expression in bisexual flowers, indicating that they may play a key role in the sex determination process of *C. retusus*. In summary, our efforts not only increase the existing knowledge of the *MADS-box* gene family within C. retusus, but also provide a foundation for further exploration of the flower evolution and gender determination mechanisms of this species.

## Method

### Plant materials

The experimental materials used for transcriptome sequencing were obtained from three *C. retusus* varieties (‘XueZaoHua’ or ‘XZH’, ‘XueDengLong’ or ‘XDL’, and ‘XueXuan’ or XX) grown at the experimental field of Shandong Agricultural University (36°10′ N, 117°9′ W). In mid-April 2023, flower organs were collected at four different development stages, including the floral bud stage (B), initial flowering stage (I), full flowering stage (Full), and final flowering stage (Final). Sample each variety three times for each period to obtain three replicates. These samples were rapidly frozen in liquid nitrogen and stored at -80℃ for subsequent transcriptome sequencing.

Materials from an additional eight *C. retusus* varieties (‘XueLuo’ or ‘XL’, 'BaoMa' or ‘BM’, 'H-19–61' or ‘H19’, 'XueRongQiu' or ‘XRQ’, 'XueLi' or ‘XI’, 'NiuYe' or ‘NY’, 'T-8', and 'MuZhu' or ‘MZ’) were also collected from the same location and during the same period mentioned earlier. The entire flower organs at the initial flowering stage of these varieties were chosen as the experimental materials. A portion of the collected materials was rapidly frozen in liquid nitrogen and stored at -80℃ for subsequent qRT-PCR experiments. Another portion was subjected to vertical sectioning using a razor blade and observed under a stereomicroscope to document the growth conditions of the pistils and stamens.

Among the aforementioned 11 *C. retusus* varieties, XZH, XI, NY, T-8, and MZ are hermaphroditic flower varieties, meaning they only have the hermaphroditic flowers. On the other hand, XDL, XX, XL, BM, H19, and XRQ are male flower varieties, indicating that they produce only male flowers. These 11 varieties are all clones independently selected by our laboratory, and all variety names and abbreviations only represent our numbering of clones. These clones are all derived from germplasm resources in different regions of China, such as Shandong Province, Henan Province, and Beijing City, and propagated through grafting. These clones only represent individuals of different genders and have been planted in the germplasm resource garden of our forestry station located at Shandong Agricultural University (36°10′ N, 117°9′ W) for scientific research and teaching purposes for a long time. Therefore, they have not been submitted to the herbarium. We regularly maintain these plants to ensure their health and stability, and to ensure reliable experimental materials can be provided.

### Identification of the *MIKC-type MADS-box* gene in *C. retusus*

The reference genome information of the *C. retusus* is based on the latest sequencing and assembly of the ‘XZH’ variety genome in our past study (unpublished, if you need it, you can contact the corresponding author). Hidden Markov Model (HMM) profile for the MADS-box (SRF-TF: PF00319) and K-box (K-box: PF01486) domains were downloaded from the Pfam database (pfam-legacy.xfam.org/) [54]. Hmmersearch was conducted using the graphical interactive software SPDE v2.0 to search for protein sequences containing the MADS-box and K-box domains in the *C. retusus* genome [55]. The obtained protein sequences, along with the 108 MADS-box protein sequences from *Arabidopsis*, were used to construct a phylogenetic tree using MEGA X software [56]. Based on the classification of type I and type II MADS-box proteins in *Arabidopsis*, it is possible to distinguish between type I and type II MADS-box proteins in the *C. retusus*. In this case, all the type II MADS-box proteins can be retained for subsequent analysis. All corresponding *MADS-box* genes are named based on their location on the chromosome. Additionally, the physicochemical properties of MADS-box proteins, including molecular weight (MW), theoretical isoelectric point (pI), instability index (II), and grand average of hydropathicity (GRAVY), be calculated using the ExPasy serve (web.expasy.org/protparam/).

### Phylogenetic and genetic structure analysis

The ClustalW algorithm was used to align the full-length sequences of 61 MIKC-type MADS-box proteins from the *C. retusus* and 45 MIKC-type MADS-box proteins from *Arabidopsis*, all parameters were set to default values. Subsequently, a maximum-likelihood phylogenetic analysis was performed under the Jones-Taylor-Thornton amino acid substitution model in MEGA X with 1000 bootstrap replicates, and the resulting tree was visualized with Evolview (evolgenius.info/evolview/) [57].

Extract the annotation information of all 61 *MIKC-type MADS-box* genes from the *C. retusus* genome annotation file, and visualize the exon–intron distribution of all genes using the Tbtools [58].

### Protein conservation domain and motif analysis of CrMADSs

Protein conservation domain analysis can be performed using the CD-Search tool of NCBI (ncbi.nlm.nih.gov/Structure/cdd/wrpsb.cgi). All CrMADS protein sequences be submitted to the CD-Search website to obtain information about the location of conservation domains [59].

The conserved motif in the proteins were analyzed using the MEME (meme-suite.org/meme/tools/meme) databased [60]. The CrMADS sequences are submitted, and the following parameters are used for the analysis: classic mode, allowing zero or one occurrence per sequence, and a total number of motifs set to 20. Finally, visualize the distribution of all structural domains and motifs using Python.

### Analysis of promoter *cis*-acting element of *CrMADSs*

The 2000 bp upstream of the start codon of 61 *CrMADS* genes was extracted, using the SPDE v2.0 software. Subsequently, all the sequences were submitted to the PlantCARE (http://bioinformatics.psb.ugent.be/webtools/plantcare/html/) database for prediction of cis-acting elements [40]. Based on the functional annotation of the elements, the specific elements were classified into five categories: growth-related elements, hormone-responsive elements, light-responsive elements, stress- responsive elements, and transcription factor binding sites. Finally, the number of specific elements was counted for visualization, and the distribution of elements was visualized using Tbtools.

### Genomic collinearity analysis of *CrMADS* genes

To perform intra-species genome collinearity analysis of the *C. retusus*, MCScanX was used to identify all collinear blocks within the *C. retusus* genome [61]. The downstream analysis software of MCScanX, “detect_collinearity_within_gene_families”, was utilized to further analyze the collinear pairs of *CrMADS* genes that have a collinear relationship. Finally, Circos was used to display the collinearity information.

To analyze the collinearity relationship of *MADS-box* genes between the *C. retusus* and two other Oleaceae plants, *Olea europaea* and *Syringa oblata*, the reference genomes of *O. europaea* and *S. oblata* were downloaded [29, 30]. The same method as mentioned above was used to identify the genomic collinearity information and collinear *MADS-box* gene pairs. Finally, the Dual Systeny Plot for MCScanX feature in Tbtools was employed for visualization. The Ka/Ks values between all collinear *CrMADS* gene pairs were calculated based on the CDS sequence and amino acid sequence, and were calculated by Simple Ka/Ks Calculator of TBtools.

### Expression level analysis

Fragments Per Kilobase Million (FPKM) values of 61 *CrMADS* genes were extracted from the previous transcriptome data (unpublished), and a heatmap was generated to display the data, the data used to construct a heatmap is obtained by taking the average of three replicates. The data in the heatmap were normalized by row using the ZeroToOne method, and row clustering was performed on the normalized data.

For qRT-PCR, total RNA from the materials at the initial flowering stage of the eight mentioned varieties was extracted using the Trizol method, and then quality-checked on a NanoDrop One UV spectrophotometer (Thermo Scientific, USA). Subsequently, the first-strand cDNA was synthesized using the Evo M-MLV Plus 1st Strand cDNA Synthesis Kit AG11615 (AG). The qRT-PCR was performed on the CFX-96 real-time PCR detection system (Bio-Rad, USA). Each experiment consisted of three independent biological replicates, with three technical replicates per sample. The UBIQUITIN CARRIER PROTEIN2 (UBC2) gene (Table S1) of *C. retusus* was used as an internal control. Relative expression levels of each target gene were analyzed the 2^−∆∆CT^ method [62]. The primers used are shown in Table S1.

### Supplementary Information

Supplementary Material 1.

Supplementary Material 2.

## Data Availability

The genomic information of the *Chionanthus retusus* mentioned in the article is available from the corresponding author upon reasonable request. This assembly used HiC and PacBio methods, with a scaffold quantity of 134. The Hi-C and ONT data, as well was the assemblies have been deposited to National Genomics Data Center with Bioproject ID of CRA011999. Accession "CRA011999" was publicly disclosed on June 20, 2024.

## Abbreviations

Ks: Synonymous
Ka: Non-synonymous
bp: Base pair
FPKM: Fragments per Kilobase Million
MW: Molecular weight
pI: Isoelectric point
II: Instability index
GRAVY: Grand average of hydropathicity

## References

[1] Bretzel F (2016). Wildflowers: From conserving biodiversity to urban greening—A review. Urban Forestry & Urban Greening.

[2] Ren Y (2017). Enhancing plant diversity and mitigating BVOC emissions of urban green spaces through the introduction of ornamental tree species. Urban Forestry & Urban Greening.

[3] Meng M (2022). Evaluating the Seasonal Change of Temperature on Shrub Seeds in Landscape Plan. Iranian Journal of Science and Technology, Transactions of Civil Engineering.

[4] Atchudan R (2017). Facile green synthesis of nitrogen-doped carbon dots using Chionanthus retusus fruit extract and investigation of their suitability for metal ion sensing and biological applications. Sens Actuators, B Chem.

[5] He Y (2017). Characterization of the complete chloroplast genome of Chinese fringetree (Chionanthus retusus). Conserv Genet Resour.

[6] Song J-H, Oak M-K, Hong S-P (2016). Morphological traits in an androdioecious species, Chionanthus retusus (Oleaceae). Flora.

[7] Park S (2022). Relationship between Leaf Traits and PM-Capturing Capacity of Major Urban-Greening Species. Horticulturae.

[8] Song J-H, Hong S-P (2020). Identity and localization of floral scent components in an androdioecious species, Chionanthus retusus (Oleaceae). Journal of Asia-Pacific Biodiversity.

[9] Vernet P (2016). Evidence for the long-term maintenance of a rare self-incompatibility system in Oleaceae. New Phytol.

[10] Wolf DE (2001). Sex Determination in the Androdioecious Plant Datisca glomerata and Its Dioecious Sister Species D. cannabina. Genetics.

[11] Zhou X-J, Ma L, Liu W-Z (2016). Functional androdioecy in the rare endemic tree Tapiscia sinensis. Bot J Linn Soc.

[12] Duan Y (2019). Functional androdioecy in the ornamental shrub Osmanthus delavayi (Oleaceae). PLoS ONE.

[13] Pannell JR, Jordan CY (2022). Evolutionary Transitions Between Hermaphroditism and Dioecy in Animals and Plants. Annual Review of Ecology, Evolution and Systematics..

[14] Charlesworth D (2008). Androdioecy and the evolution of dioecy. Biological Journal of the Linnean Society..

[15] Soejima A, Maki M, Ueda K. Genetic variation in relic and isolated populations of Chionanthus retusus(Oleaceae) of Tsushima Island and the Tôno region. Japan Genes & Genetic Systems. 1998;73(1):29–37.

[16] Par̆enicová L (2003). Molecular and Phylogenetic Analyses of the Complete MADS-Box Transcription Factor Family in Arabidopsis : New Openings to the MADS World[W]. Plant Cell.

[17] Becker A, Theißen G (2003). The major clades of MADS-box genes and their role in the development and evolution of flowering plants. Mol Phylogenet Evol.

[18] Kwantes M, Liebsch D, Verelst W (2011). How MIKC* MADS-Box Genes Originated and Evidence for Their Conserved Function Throughout the Evolution of Vascular Plant Gametophytes. Mol Biol Evol.

[19] Smaczniak C (2012). Developmental and evolutionary diversity of plant MADS-domain factors: insights from recent studies. Development.

[20] Theißen G, Melzer R, Rümpler F (2016). MADS-domain transcription factors and the floral quartet model of flower development: linking plant development and evolution. Development.

[21] Ditta G (2004). The SEP4 Gene of Arabidopsis thaliana Functions in Floral Organ and Meristem Identity. Curr Biol.

[22] Favaro R (2003). MADS-Box Protein Complexes Control Carpel and Ovule Development in Arabidopsis. Plant Cell.

[23] Kaufmann K, Melzer R, Theissen G (2005). MIKC-type MADS-domain proteins: structural modularity, protein interactions and network evolution in land plants. Gene.

[24] Gramzow L, Theissen G (2010). A hitchhiker's guide to the MADS world of plants. Genome Biol.

[25] Puranik S (2014). Structural Basis for the Oligomerization of the MADS Domain Transcription Factor SEPALLATA3 in Arabidopsis. Plant Cell.

[26] Yang Y, Fanning L, Jack T (2003). The K domain mediates heterodimerization of the Arabidopsis floral organ identity proteins, APETALA3 and PISTILLATA. Plant J.

[27] Dreni L (2011). Functional Analysis of All AGAMOUS Subfamily Members in Rice Reveals Their Roles in Reproductive Organ Identity Determination and Meristem Determinacy. Plant Cell.

[28] Smaczniak C (2012). Characterization of MADS-domain transcription factor complexes in Arabidopsis flower development. Proc Natl Acad Sci.

[29] Ma B (2022). Lilac (Syringa oblata) genome provides insights into its evolution and molecular mechanism of petal color change. Communications Biology.

[30] Rao G (2021). De novo assembly of a new Olea europaea genome accession using nanopore sequencing. Horticulture Research.

[31] Zhang L (2020). The ancient wave of polyploidization events in flowering plants and their facilitated adaptation to environmental stress. Plant, Cell Environ.

[32] Liu M (2019). Genome-wide investigation of the MADS gene family and dehulling genes in tartary buckwheat (Fagopyrum tataricum). Planta.

[33] Zhang L (2017). Genome-wide identification, characterization of the MADS-box gene family in Chinese jujube and their involvement in flower development. Sci Rep.

[34] Wei X (2015). Genome-wide identification and analysis of the MADS-box gene family in sesame. Gene.

[35] Rümpler F, et al. The Origin of Floral Quartet Formation—Ancient Exon Duplications Shaped the Evolution of MIKC-type MADS-domain Transcription Factor Interactions. Mol Biol Evol. 2023;40(5):msad088. 10.1093/molbev/msad088 PMC10152394 37043523

[36] Hernández-Hernández T, Martínez-Castilla LP, Alvarez-Buylla ER (2006). Functional Diversification of B MADS-Box Homeotic Regulators of Flower Development: Adaptive Evolution in Protein-Protein Interaction Domains after Major Gene Duplication Events. Mol Biol Evol.

[37] Vandenbussche M (2003). Structural diversification and neo-functionalization during floral MADS-box gene evolution by C-terminal frameshift mutations. Nucleic Acids Res.

[38] Shan H (2007). Patterns of gene duplication and functional diversification during the evolution of the AP1/SQUA subfamily of plant MADS-box genes. Mol Phylogenet Evol.

[39] Himani S (2014). Computational analysis of cis-acting regulatory elements in 5 ' regulatory regions of sucrose transporter gene families in wheat and Arabidopsis. Research Journal of Biotechnology.

[40] Lescot M (2002). PlantCARE, a database of plant cis-acting regulatory elements and a portal to tools for in silico analysis of promoter sequences. Nucleic Acids Res.

[41] Ó’Maoiléidigh DS (2013). Control of Reproductive Floral Organ Identity Specification in Arabidopsis by the C Function Regulator AGAMOUS. Plant Cell.

[42] Fourquin C, Ferrándiz C (2012). Functional analyses of AGAMOUS family members in Nicotiana benthamiana clarify the evolution of early and late roles of C-function genes in eudicots. Plant J.

[43] Lin Z (2020). Genome-wide identification of MADS-box gene family in sacred lotus (Nelumbo nucifera) identifies a SEPALLATA homolog gene involved in floral development. BMC Plant Biol.

[44] Schilling S (2020). Genome-wide analysis of MIKC-type MADS-box genes in wheat: pervasive duplications, functional conservation and putative neofunctionalization. New Phytol.

[45] Xie L (2021). TaVrt2, an SVP-like gene, cooperates with TaVrn1 to regulate vernalization-induced flowering in wheat. New Phytol.

[46] Voogd C (2022). A MADS-box gene with similarity to FLC is induced by cold and correlated with epigenetic changes to control budbreak in kiwifruit. New Phytol.

[47] Montiel G, et al. Overexpression of MADS-box Gene AGAMOUS-LIKE 12 Activates Root Development in Juglans sp. and Arabidopsis thaliana. Plants-Basel. 2020;9(4):444. 10.3390/plants9040444 PMC7238194 32252382

[48] Zhang R (2013). Disruption of the petal identity gene APETALA3-3 is highly correlated with loss of petals within the buttercup family (Ranunculaceae). Proc Natl Acad Sci.

[49] Zhang Y (2011). Functional Analysis of the Two Brassica AP3 Genes Involved in Apetalous and Stamen Carpelloid Phenotypes. PLoS ONE.

[50] Zheng Y (2009). Global Identification of Targets of the Arabidopsis MADS Domain Protein AGAMOUS-Like15. Plant Cell..

[51] Zheng Q, Zheng Y, Perry SE (2013). AGAMOUS-Like15 Promotes Somatic Embryogenesis in Arabidopsis and Soybean in Part by the Control of Ethylene Biosynthesis and Response. Plant Physiol..

[52] Dreni L, Kater MM (2014). MADS reloaded: evolution of the AGAMOUS subfamily genes. New Phytol.

[53] Kramer EM, Jaramillo MA, Di Stilio VS (2004). Patterns of Gene Duplication and Functional Evolution During the Diversification of the AGAMOUS Subfamily of MADS Box Genes in Angiosperms. Genetics.

[54] Finn RD, Clements J, Eddy SR (2011). HMMER web server: interactive sequence similarity searching. Nucleic Acids Res..

[55] Xu D (2021). SPDE: A Multi-functional Software for Sequence Processing and Data Extraction. Bioinformatics..

[56] Kumar S (2018). MEGA X: Molecular Evolutionary Genetics Analysis across Computing Platforms. Mol Biol Evol.

[57] Subramanian B (2019). Evolview v3: a webserver for visualization, annotation, and management of phylogenetic trees. Nucleic Acids Res.

[58] Chen C (2020). TBtools: An Integrative Toolkit Developed for Interactive Analyses of Big Biological Data. Mol Plant.

[59] Marchler-Bauer A, Bryant SH (2004). CD-Search: protein domain annotations on the fly. Nucleic Acids Res..

[60] Bailey TL (2009). MEME SUITE: tools for motif discovery and searching. Nucleic Acids Res..

[61] Wang Y (2012). MCScanX: a toolkit for detection and evolutionary analysis of gene synteny and collinearity. Nucleic Acids Res.

[62] Livak KJ, Schmittgen TD (2001). Analysis of relative gene expression data using real-time quantitative PCR and the 2(-Delta Delta C(T)) Method. Methods.

