# Supplementary material for: Genome-wide identification and expression pattern analysis of MIKC-Type MADS-box genes in Chionanthus retusus, an androdioecy plant
Source: BMC Genomics. 2024 Jul 2;25:662. doi: 10.1186/s12864-024-10569-8 (PMC11220994; doi:10.1186/s12864-024-10569-8)
Supplement: Supplementary file 1 — Supplementary Material 1. [file 12864_2024_10569_MOESM1_ESM.pdf]

Genome-Wide Identification and Expression Pattern Analysis of *MIKC-Type MADS-box* Genes in *Chionanthus retusus*, an Androdioecy Plant

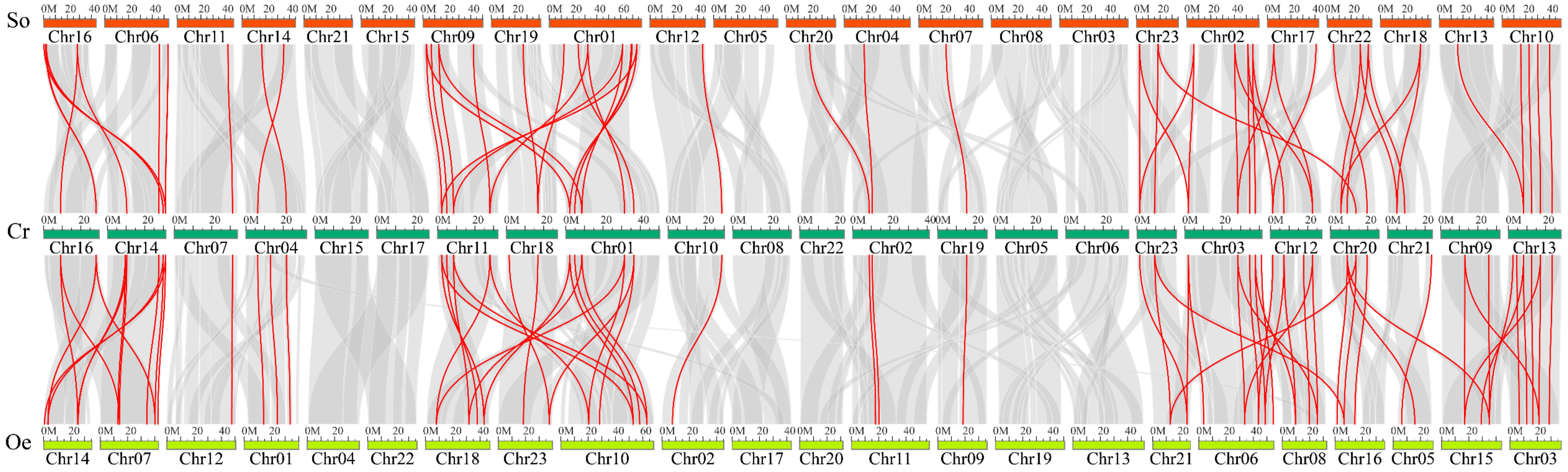

Fig. S1. Interspecific genomic collinearity of *MADS* genes. ‘So’ represents the chromosome of *Syringa oblata*, ‘Cr’ represents the chromosome of *C. retusus*, and ‘Oe’ represents the chromosome of *Olea europaea*. The red line represents a collinear *MADS* gene pair, while the gray ribbon represents a collinear segment of the chromosome. The intersection point between each red line and the chromosome represents the position of the corresponding *MADS-box* gene.

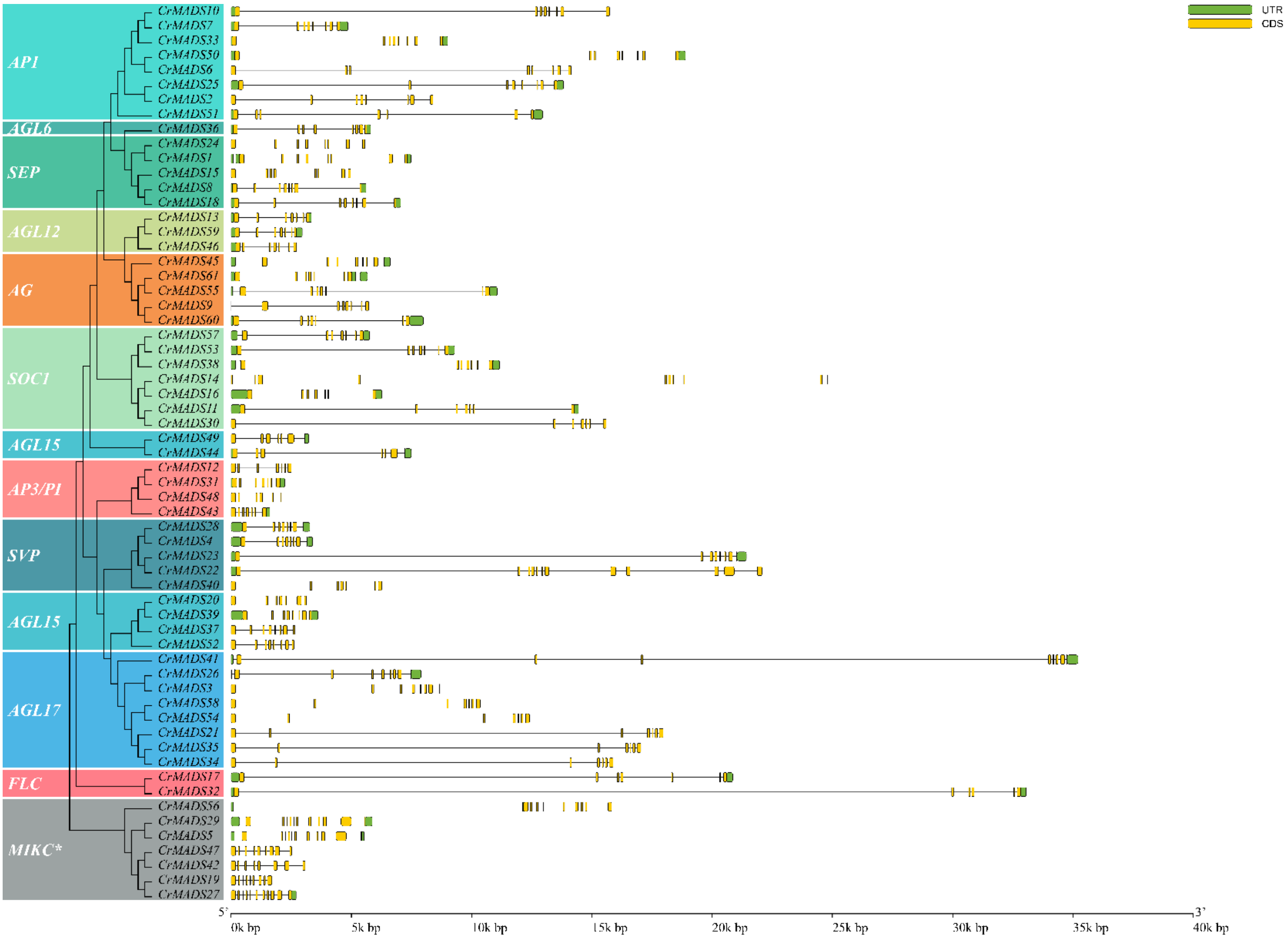

Fig. S2. Phylogenetic relationships and gene structure of *CrMADSs*.

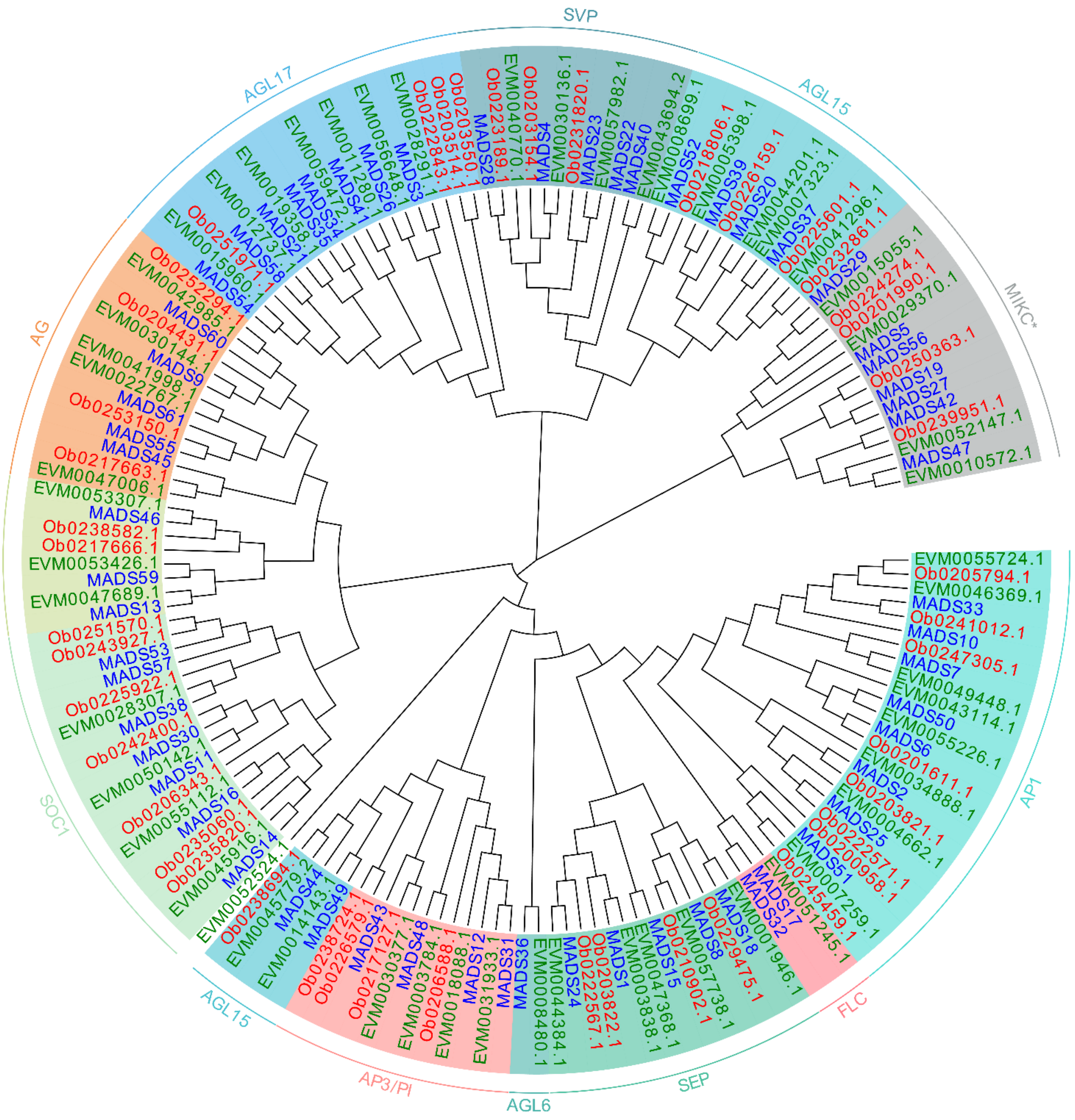

Fig. S3. Phylogenetic analysis of *MADS-box* genes in *C. retusus*, *O. europaea*, and *S. oblata*. Among them, the blue font for the *C. retusus MADS-box* genes, the red font for the *S. oblata MADS-box* genes, and the green font for the *O. europaea MADS-box* genes.
